# St. Louis Medical Journal

**Published:** 1850-06

**Authors:** 


### ST. LOUIS MEDICAL JOURNAL.

We inadvertantly omitted an acknowledgment of the revival of this work in our former number. It was suspended by the great fire in St. Louis, but we hope it may now go on rejoicing in strength and usefulness. The only change that has been made in the editorial department is in the substitution of the name of Dr. John B. Johnson, as one of the editors, in place of Dr. Joseph N. McDowell. We formed a very favorable opinion of the new editor during our attendance at the recent meeting of the National Association of Physicians.

